# Is Less More? A Meta-Analysis of Non-Intubated Versus Intubated VATS for Anatomic Resections in Non-Small Cell Lung Cancer

**DOI:** 10.3390/jcm14196731

**Published:** 2025-09-24

**Authors:** Dimitrios E. Magouliotis, Anna P. Karamolegkou, Prokopis-Andreas Zotos, Fabrizio Minervini, Ugo Cioffi, Marco Scarci

**Affiliations:** 1 Department of Cardiac Surgery Research, Lankenau Institute for Medical Research, Wynnewood, PA 19096, USA; dimitrios.magouliotis.18@alumni.ucl.ac.uk; 2 Department of Anesthesiology, Hippocration General Hospital of Athens, 11527 Athens, Greece; karamolegkoua.97@gmail.com; 3 Department of Cardiothoracic Surgery, University of Thessaly, Biopolis, 41110 Larissa, Greece; zotospro@hotmail.com; 4 Luzern Kanton Hospital, 6003 Luzern, Switzerland; fabriziominervini@hotmail.com; 5 Department of Surgery, University of Milan, 20122 Milan, Italy; ugo.cioffi@guest.unimi.it; 6 Department of Cardiothoracic Surgery, Hammersmith Hospital, Imperial College Healthcare, National Health Service (NHS) Trust, London W2 1NY, UK

**Keywords:** lung cancer, non-small cell lung cancer (NSCLC), video-assisted thoracoscopic surgery (VATS), non-intubated VATS (NIVATS), awake VATS, lobectomy

## Abstract

**Objective:** Non-intubated video-assisted thoracoscopic surgery (NIVATS) has emerged as a less invasive alternative to conventional intubated VATS (IVATS) for patients undergoing lobectomy for non-small cell lung cancer (NSCLC). However, concerns regarding its safety, efficacy, and oncologic adequacy remain. This meta-analysis aimed to compare perioperative and short-term outcomes between NIVATS and IVATS. **Methods:** A systematic review and meta-analysis were conducted in accordance with PRISMA guidelines. PubMed, Scopus, and Cochrane CENTRAL were searched through 30 June 2025. Studies comparing NIVATS and IVATS for anatomical lung resections (lobectomy and/or segmentectomy) in NSCLC were included; wedge resections were excluded. Primary endpoints included postoperative complications, operative time, intraoperative blood loss, lymph node yield, and 30-day mortality. Secondary endpoints were chest tube duration, hospital length of stay, anesthetic time, and conversion to thoracotomy rates. Risk of bias was assessed primarily with ROBINS-I; the Newcastle–Ottawa Scale was applied for sensitivity. **Results:** A total of seven studies (six retrospective and one randomized controlled trial) encompassing 851 patients (374 NIVATS, 477 IVATS) were included. NIVATS was associated with a significantly lower rate of postoperative complications (OR 0.50; 95% CI: 0.30–0.86; *p* = 0.01; *I*^2^ = 0%), shorter operative time (minutes) (WMD −21.85; 95% CI: −38.49, −5.21; *p* = 0.01), anesthetic time (minutes) (WMD −4.62; 95% CI: −6.60, −2.65; *p* < 0.01), and reduced intraoperative blood loss (mL) (WMD −24.36; 95% CI: −30.67, −18.05; *p* < 0.01). There were no significant differences in lymph node yield or conversion to thoracotomy rates. No 30-day mortality was reported in either group. The quality of included studies was moderate, and publication bias was not evident. **Conclusions:** NIVATS appears to be a safe and effective alternative to IVATS in selected patients undergoing lobectomy for NSCLC. It offers improved perioperative outcomes without compromising surgical or oncologic standards. Prospective trials are needed to confirm these findings and assess long-term survival.

## 1. Introduction

Lung cancer remains a leading cause of cancer-related mortality worldwide, with surgical resection continuing to be the cornerstone of treatment for early-stage non-small-cell lung cancer (NSCLC) [1,2]. Video-assisted thoracoscopic surgery (VATS) has largely supplanted open thoracotomy due to its association with less postoperative pain, reduced hospital stay, and fewer complications [3,4]. Traditionally, VATS is performed under general anesthesia with endotracheal intubation and single-lung ventilation. While this technique offers optimal surgical conditions, it carries well-documented risks, particularly in elderly patients and those with compromised cardiopulmonary function. These risks include ventilator-induced lung injury, airway trauma, diaphragmatic dysfunction, and prolonged recovery [5,6].

In recent years, the use of non-intubated VATS (NIVATS), also known as awake or tubeless thoracic surgery, has gained traction as a potentially less invasive alternative [7,8]. By avoiding intubation, muscle relaxants, and mechanical ventilation, NIVATS may mitigate some of the physiologic stress associated with conventional anesthesia. Studies have suggested that NIVATS may be associated with shorter operating room times, reduced chest tube duration, decreased hospital length of stay, and similar rates of postoperative complications [9]. However, concerns remain regarding the feasibility and oncologic adequacy of NIVATS, especially in the context of anatomic lung resections such as lobectomy. While NIVATS may reduce ventilator-associated lung injury, diaphragmatic dysfunction, and recovery time, it carries risks including hypoxemia, hypercapnia, coughing or movement during surgery, and need for conversion to intubation or thoracotomy. Previous exploratory reviews have hinted at the equivalence of non-intubated and intubated approaches in terms of short-term outcomes, yet high-quality evidence remains limited and fragmented [10,11,12]. Moreover, recent studies have begun to explore longer-term outcomes such as survival, which are particularly relevant in oncologic surgery. In this meta-analysis, we aim to synthesize the current evidence comparing awake (non-intubated) versus traditional (intubated) VATS (IVATS) in patients undergoing lung resection for cancer. Importantly, the current evidence base for anatomical resections, particularly lobectomy, in NSCLC is derived predominantly from observational studies, which are heterogeneous in design and reporting. As a result, uncertainty remains regarding the oncologic adequacy of NIVATS, including the completeness of lymph node station sampling, achievement of R0 resection margins, and accurate pathologic upstaging. This review focuses on adult patients with NSCLC undergoing anatomical resection (lobectomy ± segmentectomy). We focus on perioperative morbidity, surgical efficiency, postoperative recovery, and oncologic outcomes, to provide a comprehensive and updated evaluation of this evolving technique.

## 2. Materials and Methods

### 2.1. Search and Articles Selection Strategy

This meta-analysis was conducted according to a predefined protocol agreed upon by all contributing authors and adhered to the Preferred Reporting Items for Systematic Reviews and Meta-Analyses (PRISMA) and Meta-analyses Of Observational Studies in Epidemiology (MOOSE) guidelines [13,14]. The protocol was registered in the Open Science Framework (OSF) Registries (registration doi: https://doi.org/10.17605/OSF.IO/RM7FX). A comprehensive literature search was carried out using three electronic databases: PubMed (MEDLINE), Scopus (Elsevier), and the Cochrane Central Register of Controlled Trials (CENTRAL). The final search was performed on 30 June 2025. ClinicalTrials.gov, ICTRP, conference abstracts, and forward citation searches (Google Scholar “cited by”) were included.

The search strategy included combinations of the following keywords and MeSH terms: “awake VATS,” “non-intubated VATS,” “tubeless thoracic surgery,” “video-assisted thoracic surgery,” “general anesthesia,” “lung cancer,” and “non-small cell lung cancer (NSCLC)” (Table S1). Boolean operators were used to optimize sensitivity and specificity of the search. Studies were considered eligible if they met the following criteria:

1. Original articles including more than 10 patients;
2. Published between 1 January 2010, and 30 June 2025;
3. Written in English;
4. Involving human subjects;
5. Comparing awake (non-intubated) VATS to conventional (intubated) VATS in patients undergoing pulmonary anatomical resection (defined as lobectomy ± segmentectomy) for lung cancer; wedge resections were excluded, as they are non-anatomical procedures with limited oncologic comparability, potentially introducing heterogeneity into the analysis; and
6. Reporting at least one perioperative or oncologic outcome (e.g., operative time, chest tube duration, length of stay, complications, survival).

We restricted to 2010 onwards to capture contemporary adoptions of NIVATS; no pivotal pre-2010 studies were identified. Eligible studies included prospective or retrospective comparative cohorts, propensity-matched studies, and RCTs. Single-arm series were excluded. Duplicates were removed, and the references of included studies were manually reviewed to identify any additional eligible articles. When duplicate populations were suspected, the most complete or recent dataset was included. Data extraction was independently conducted by two reviewers (DEM, APK) using a standardized form. Discrepancies in study selection or data extraction were resolved by discussion, and when needed, adjudicated by a senior author (MS) until consensus was reached.

### 2.2. Data Extraction and Endpoints

Two independent reviewers (DEM and APK) extracted data from all eligible studies using a standardized collection form. Discrepancies were resolved by consensus or through adjudication by a senior author (MS). For each study, the following baseline characteristics were collected: first author, year of publication, country of origin, sample size, patient demographics, and type of surgical procedure performed. Authors would be contacted in the case of missing data.

The primary endpoints of this meta-analysis were the incidence of postoperative complications, defined as any surgery- or anesthesia-related adverse event occurring during hospitalization or within 30 days postoperatively, and 30-day all-cause mortality. Complication data were extracted in aggregate and, where available, classified by type, including atelectasis, pneumonia, prolonged air leak, cardiac arrhythmia, among others.

Secondary endpoints included mean operative time (MOT; measured in minutes), intraoperative blood loss (in milliliters), chest tube duration (in days), length of hospital stay (in days), conversion rate to general anesthesia or thoracotomy, readmission rate, and overall survival when available. Outcomes were recorded separately for the non-intubated (NIVATS) and intubated (IVATS) groups. When results were presented as medians and interquartile ranges, estimates of means and standard deviations were calculated using validated statistical methods. Where studies reported only proportions or incomplete data, corresponding authors were to be contacted. Statistical analyses were performed in Review Manager (RevMan) 5.4.1. (The Cochrane Collaboration, London, UK, 2020).

### 2.3. Sensitivity Analysis on Survival Endpoints

The leave-one-out method involves performing a meta-analysis on each subset of the studies obtained by leaving out exactly one study.

### 2.4. Quality and Publication Bias Assessment

The methodological quality of the included studies was assessed independently by two reviewers (DEM and APK) using both the ROBINS-I tool (Risk Of Bias In Non-randomized Studies—of Interventions) [15] and the Newcastle-Ottawa Scale (NOS) [16] for observational studies. We employed the ROBINS-I tool as the primary tool for risk-of-bias assessment, with NOS applied as a supplementary descriptor for sensitivity. The ROBINS-I tool was used to evaluate seven domains of potential bias: confounding, selection of participants, classification of interventions, deviations from intended interventions, missing data, measurement of outcomes, and selection of the reported result. Each domain was rated as low, moderate, serious, or critical risk, and an overall risk of bias judgment was assigned accordingly. Discrepancies in judgment were resolved through discussion or referral to a third reviewer (MS).

In addition, the NOS was employed to assess study quality based on three categories: selection of study groups, comparability of groups, and ascertainment of exposure or outcomes. Studies scoring 7 to 9 points were considered high quality, those scoring 4 to 6 points were of moderate quality, and those scoring below 4 were considered low quality.

Publication bias was assessed visually using funnel plots for the primary outcome and, when sufficient studies were available (≥10), quantitatively using Egger’s regression test. A symmetrical funnel plot and a non-significant *p*-value (*p* > 0.05) were interpreted as low risk of publication bias.

## 3. Results

### 3.1. Search Strategy and Patient Demographics

**Figure 1:**
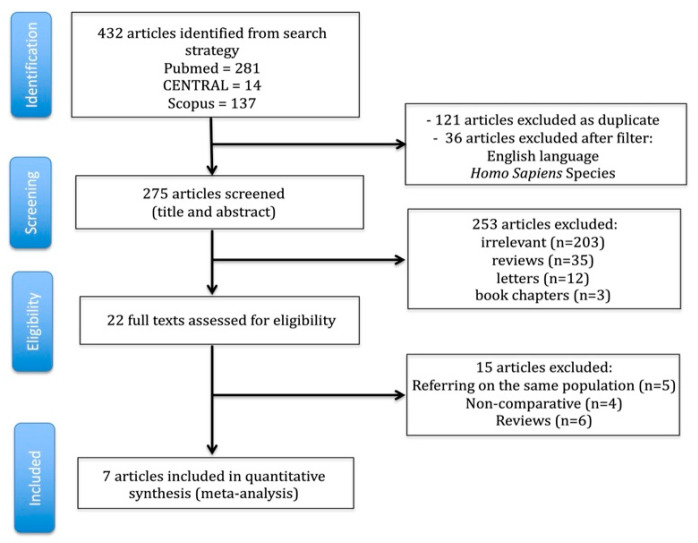
Trial flow of the current meta-analysis.

The flow diagram detailing the literature search and study selection process is presented in Figure 1, while the PRISMA checklist is available in Table S2. The characteristics of the included studies are summarized in Table 1. From an initial pool of 432 articles, seven studies [9,17,18,19,20,21,22] met the inclusion criteria and were incorporated into both the qualitative and quantitative analyses. The inter-reviewer agreement for study inclusion was classified as “almost perfect” (κ = 0.881; 95% CI: 0.734–1.000).

Six studies [9,17,18,19,21,22] were retrospective cohort analyses comparing outcomes between NIVATS and conventional IVATS, one study used propensity score matching methodology [18], and one study [20] was a randomized controlled trial (RCT). The studies were conducted in Saudi Arabia [17], China [9,19,20,21], the United States [18], and Thailand [22], and were published between 2011 and 2024. Sample sizes across studies ranged from 60 to 216 patients, with a total pooled population of 851 patients, comprising 374 patients in the NIVATS group and 477 in the IVATS group. Baseline demographic characteristics and clinical profiles were generally well balanced across the included cohorts. Five studies [9,17,19,21,22] included only patients undergoing VATS lobectomy and two studies [18,20] included patients undergoing either VATS lobectomy or sublobar resections. Pulmonary lobectomy was carried out using either a three-port or four-port thoracoscopic approach. The patient was placed in the lateral decubitus position for optimal access. Endoscopic staplers were employed to divide the pulmonary vessels, bronchus, and lung parenchyma. The excised lobe was retrieved using a specimen bag.

In the NIVATS group, anesthesia was typically delivered using a combination of regional and monitored sedation techniques to maintain spontaneous ventilation while ensuring adequate analgesia and patient immobility. Most studies employed thoracic epidural anesthesia or intercostal nerve blocks, often supplemented with intrathoracic vagal blockade to suppress cough reflex. Sedation was provided using agents such as dexmedetomidine, propofol, or remifentanil, titrated to maintain patient comfort without compromising spontaneous breathing. Oxygen was administered via facemask, high-flow nasal cannula, or laryngeal mask airway, depending on institutional protocols. This approach allowed for avoidance of muscle relaxants, endotracheal intubation, and mechanical ventilation, thereby reducing airway trauma and ventilator-associated complications.

Perioperative outcomes including postoperative complications, operative time, intraoperative blood loss, chest tube duration, length of hospital stay, conversion to thoracotomy or general anesthesia, and 30-day mortality were reported across all included studies and are summarized in Table 2. A detailed breakdown of postoperative complications by type is provided in Table 3.

### 3.2. Primary Endpoints: Complications and Mortality

**Figure 2:**
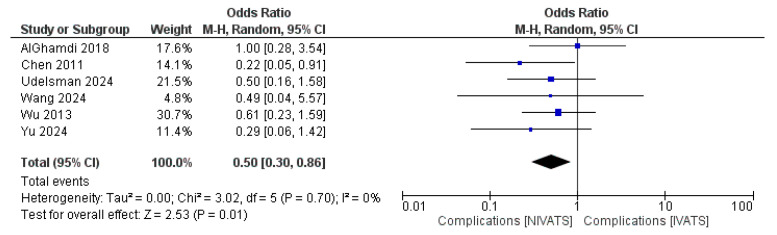
Forest plot regarding complications comparing non-intubated and intubated video-assisted thoracoscopic surgery (NIVATS and IVATS, respectively) [9,17,18,20,21,22]. Blue box: Study effect size; box size = study weight; horizontal line = 95% CI. Black diamond: Pooled effect; center = summary mean difference; width = 95% CI.

All seven studies [9,17,18,19,20,21,22] reported data on postoperative complications and mortality. The pooled incidence of complications was 17.4% (65/374) in the NIVATS group and 20.0% (95/477) in the IVATS group. The types and frequency of specific complications are detailed in Table 3, with the most common events including atelectasis, pneumonia, and prolonged air leak. All studies reported zero mortality. According to our findings, complications were significantly higher in the IVATS group (OR: 0.50 [95% CI: 0.30, 0.86]; *p* = 0.01) (Figure 2). The heterogeneity was zero regarding the complications.

### 3.3. Secondary Endpoints

**Figure 3:**
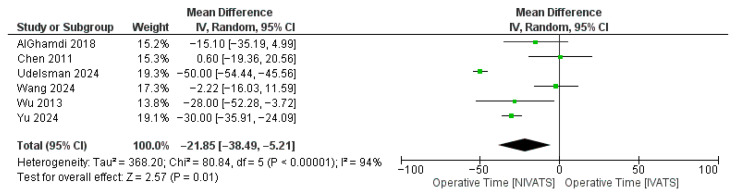
Forest plot regarding mean operative time (MOT) comparing non-intubated and intubated video-assisted thoracoscopic surgery (NIVATS and IVATS, respectively) [9,17,18,20,21,22]. Green box: Effect size of each study; box size = study weight; horizontal line = 95% CI. Black diamond: Pooled effect; center = summary mean difference; width = 95% CI.

**Figure 4:**
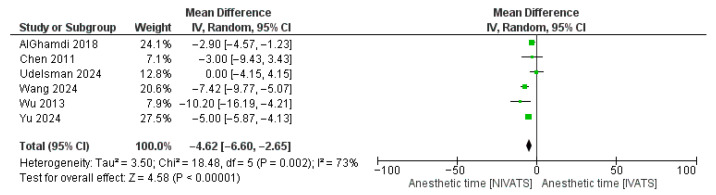
Forest plot regarding mean anesthetic time comparing non-intubated and intubated video-assisted thoracoscopic surgery (NIVATS and IVATS, respectively) [9,17,18,20,21,22]. Green box: Effect size of each study; box size = study weight; horizontal line = 95% CI. Black diamond: Pooled effect; center = summary mean difference; width = 95% CI.

Regarding the intraoperative parameters, NIVATS was superior in terms of MOT (WMD: −21.85 [95% CI: −38.49, −5.21]; *p* < 0.01) (Figure 3), anesthetic time (WMD: −4.62 [95% CI: −6.60, −2.65]; *p* < 0.01) (Figure 4), and blood loss (WMD: −24.36 [95% CI: −30.67, −18.05]; *p* < 0.01) compared to IVATS. No significant difference was reported between the two groups regarding the conversion to thoracotomy, the chest tube duration, the length of hospital stay, and the number of lymph nodes sampled. Data on conversion events were variably reported across studies. While most series reported conversion to thoracotomy, information on airway conversion to intubation was not consistently available, precluding separate pooled analysis.

### 3.4. Sensitivity Analysis and Publication Bias Assessment

**Figure 5:**
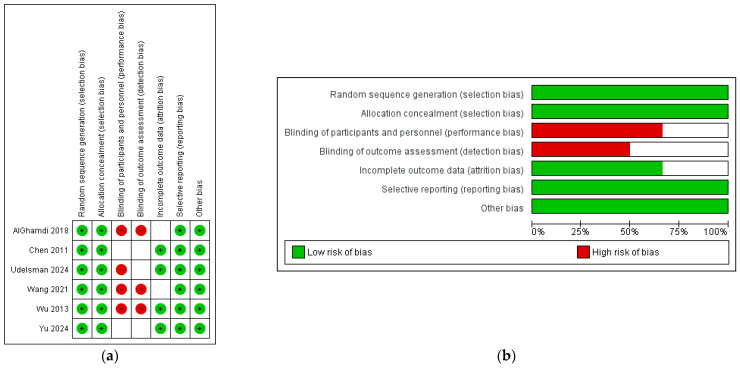
(**a**). Risk of Bias in Non-Randomized Studies of Interventions tool with traffic lights. (**b**) Risk of Bias in Non-Randomized Studies of Interventions tool with summary plot [9,17,18,20,21,22].

No difference regarding the survival outcomes was found after performing the leave-one-out sensitivity analysis. Heterogeneity was low regarding the primary endpoints. Nonetheless, heterogeneity was high regarding MOT and LOS. The assessment according to the ROBINS-I tool is demonstrated in Figure 5.

## 4. Discussion

This meta-analysis synthesizes data from seven studies comparing NIVATS to conventional IVATS in patients undergoing lung resection for non-small cell lung cancer (NSCLC). Our findings indicate that NIVATS is associated with significantly fewer postoperative complications, reduced operative and anesthetic time, and lower intraoperative blood loss, while achieving comparable oncologic adequacy. Although several outcomes reached statistical significance, the absolute effect sizes were small (e.g., ~20 min reduction in operative time, ~25 mL reduction in blood loss). These differences, while measurable, may have limited clinical impact in otherwise healthy patients. The patients included in available studies were relatively healthy, with preserved lung function and few comorbidities. In such populations, the modest perioperative differences observed may not yield substantial clinical benefit. We restricted our analysis to anatomical resections (lobectomy and segmentectomy), excluding wedge resections. This decision was made to ensure oncologic homogeneity, as wedge resections are generally considered diagnostic or palliative rather than definitive oncologic procedures. Future research should focus on higher-risk patients, such as those with advanced age, significant comorbidities, or impaired pulmonary function, who may be more likely to benefit from NIVATS.

The most notable finding of our analysis is the reduction in postoperative complications in the NIVATS group (OR 0.50; 95% CI: 0.30–0.86; *p* = 0.01), with a particularly low heterogeneity across studies (*I*^2^ = 0%). This suggests a robust effect that supports the safety profile of the non-intubated technique. Common complications such as prolonged air leak, pneumonia, and atrial fibrillation were all slightly less frequent in the NIVATS group, although the absolute differences were modest. These findings are consistent with the known physiologic advantages of NIVATS, which avoids positive-pressure ventilation and neuromuscular blockade, thereby preserving more physiologic pulmonary function and reducing airway and diaphragmatic trauma [5].

Compared to the previous meta-analysis by Prisciandaro et al. [11], which included three studies with 204 patients, our updated analysis encompasses seven studies and 851 patients, thereby providing greater statistical power. In addition, our study incorporates more recent evidence, including a 2024 randomized controlled trial, and applies stricter definitions of anatomical resection (lobectomy ± segmentectomy, excluding wedge resections), ensuring clearer surgical stratification. These methodological improvements and the expanded evidence base likely explain why our analysis demonstrated a significant reduction in postoperative complications with NIVATS, whereas the earlier study did not. Nonetheless, both analyses converge in supporting the feasibility and short-term safety of NIVATS in appropriately selected patients, while highlighting the need for larger prospective trials to assess long-term oncologic outcomes.

Regarding intraoperative parameters, our results showed that NIVATS significantly reduced operative time (WMD: −21.85 min), anesthetic time, and blood loss, outcomes likely influenced by faster induction and recovery without the need for airway manipulation or muscle relaxants. Although these findings align with prior observational data [12], high heterogeneity (*I*^2^ > 90%) warrants cautious interpretation. Notably, chest tube duration and length of stay showed no statistically significant difference between the groups, suggesting that while operative efficiency may be improved, postoperative recovery time remains similar. An important consideration is the distinction between airway conversion to intubation and surgical conversion to thoracotomy. Most included studies reported conversion to thoracotomy, usually for technical reasons such as bleeding or dense adhesions, whereas only a minority specified airway conversions due to hypoxemia, hypercapnia, or patient movement. Because reporting was inconsistent, we were unable to pool airway and surgical conversions separately. This heterogeneity in outcome definitions underscores the need for future studies to standardize reporting of conversion events, with explicit categorization of airway versus surgical reasons, as these carry different clinical implications.

Oncologic adequacy remains a critical concern in thoracic oncology. While some early studies raised doubts about the ability to perform complete lymphadenectomy under spontaneous ventilation, our analysis did not show inferiority in terms of lymph node harvesting. In fact, many included studies reported adherence to standard oncologic protocols, including systematic nodal dissection [18]. Another key strength of our meta-analysis is the inclusion of the most recent RCT in this field [20], which confirmed no detectable difference between the two approaches regarding pulmonary complications. This trial, although modest in size, reinforces the growing consensus that NIVATS can be safely extended beyond minor resections.

Several important limitations warrant consideration. First, confounding by indication is likely, as NIVATS is often offered to patients with lower baseline risk profiles or more favorable anatomy, whereas IVATS is typically reserved for higher-risk or technically complex cases. Although one study (Udelsman et al. [18]) employed propensity score matching and another (Wang et al. [19]) performed multivariable adjustment, the majority of included studies reported unadjusted estimates. When we considered adjusted data separately, the direction of effect remained consistent, but the limited number of adjusted analyses precluded a formal adjusted-only meta-analysis. Second, outcomes may be influenced by center and surgeon expertise, as NIVATS is more frequently performed in high-volume institutions with established experience. Furthermore, most included studies were retrospective, introducing potential selection bias. Although one study employed propensity matching and another applied multivariable adjustment, the majority reported unadjusted outcomes, limiting the strength of pooled estimates. Finally, a critical limitation of the current evidence is the scarcity of long-term oncologic outcomes. Most included studies reported only short-term perioperative metrics, with limited data on recurrence or survival. As such, the impact of NIVATS on oncologic adequacy and long-term prognosis remains uncertain and should be a primary focus of future prospective trials. These factors may have biased results in favor of NIVATS and should be addressed in future multicenter prospective studies.

## 5. Conclusions

This meta-analysis demonstrates that NIVATS is a safe and effective alternative to conventional IVATS for patients undergoing lobectomy for non-small cell lung cancer. NIVATS is associated with reduced operative time, lower intraoperative blood loss, and significantly fewer postoperative complications, without compromising short-term oncologic outcomes such as lymph node yield or 30-day mortality. These findings support the selective use of NIVATS in appropriately chosen patients, particularly those with higher risk for intubation-related morbidity. However, further high-quality randomized controlled trials with long-term follow-up are warranted to validate these results and better define the role of NIVATS in thoracic oncology.

## Figures and Tables

**Table 1:** Baseline characteristics of the studies and patients that were included in the meta-analysis.

Study ID, Year	Study Design	Patients, n		Females, %		FEV1		Mean Age, y ± SD		Histology, %		Pathologic Staging	Neoadjuvant Tx	NOS
		NI	I	NI	I	NI	I	NI	I	NI	I			
AlGhamdi 2018 [17]	R	30	30	67	60	95 ± 9	95 ± 17	65 ± 11	66 ± 10	ADC: 87 SCC: 13	ADC: 83 SCC: 13	I-II	NR	6
Chen 2011 [9]	R	30	30	77	57	105 ± 18	104 ± 12	58 ± 10	57 ± 10	ADC: 83	ADC: 90	I	E	6
Udelsman 2024 [18]	R-PSM	67	134	33	33	90	98	68 ± 11	68 ± 12	-	-	I	E	8
Wang 2021 [19]	R	97	97	58	59	104 ± 7	114 ± 6	60 ± 11	62 ± 12	ADC: 97	ADC: 92	IA-IB	E	6
Wang 2024 [20]	RCT	60	60	70	60	-	-	52 ± 6	51 ± 8	-	-	-	-	-
Wu 2013 [21]	R	36	48	42	44	113 ± 26	106 ± 24	73	73	ADC: 92	ADC: 83	I	E	6
Yu 2024 [22]	R	54	78	63	51	N/A	N/A	65	65	ADC: 89 SCC: 4	ADC: 92 SCC: 8	T1-T2	Given if N2	8

Abbreviations: NOS = Newcastle-Ottawa Scale; R = Retrospective; PSM = Propensity Score Matching; RCT = Randomized Controlled Trial; NI = Non-Intubated; I = Intubated; y = years; n = number; ADC = Adenocarcinoma; SCC = Squamous Cell Carcinoma; SD = Standard Deviation.

**Table 2:** Summary of the endpoints.

Categorical Outcomes	n	OR [95% CI]	*p*	Heterogeneity	
				*I* ^2^	*p*
Complications	6	0.50 [0.30, 0.86]	0.01	0%	0.70
30-day mortality	7	Not estimable	-	-	-
Conversion to thoracotomy	4	1.65 [0.30, 8.98]	0.56	0%	0.65
Continuous outcomes	n	WMD (95% CI)		*I* ^2^	*p*
MOT	6	−21.85 [−38.49, −5.21]	0.01	94%	<0.01
Anesthetic time	6	−4.62 [−6.60, −2.65]	<0.01	73%	<0.01
Blood Loss	4	−24.36 [−30.67, −18.05]	<0.01	0%	0.57
Chest tube duration	5	−0.18 [−0.45, 0.10]	0.21	50%	0.09
LOS	6	−0.57 [−1.17, 0.03]	0.06	94%	<0.01
Total Nodes Sampled	6	−0.84 [−2.29, 0.62]	0.26	86%	<0.01

Abbreviations: n = number; MOT = Mean Operative Time; LOS = Length of Stay; OR = Odds Ratio; WMD = Weighted Mean Difference; 95% CI = 95% Confidence Intervals.

**Table 3:** Summary of the complications.

Complication	NIVATS (%)	IVATS (%)
Prolonged air leak	6.3	7.8
Pneumonia	3.0	3.9
Re-intubation	1.1	1.9
Conversion to thoracotomy	0.7	1.9
Atrial fibrillation	1.9	2.9
Bleeding	0.4	1.0

## Data Availability

The data that support the findings of this study are available from the corresponding author, upon reasonable request.

## Supplementary Materials

The following supporting information can be downloaded at https://www.mdpi.com/article/10.3390/jcm14196731/s1, Table S1: Full Search Strategies; Table S2: PRISMA checklist.
