# Supplementary material for: Is Less More? A Meta-Analysis of Non-Intubated Versus Intubated VATS for Anatomic Resections in Non-Small Cell Lung Cancer
Source: J Clin Med. 2025 Sep 24;14(19):6731. doi: 10.3390/jcm14196731 (PMC12524945; doi:10.3390/jcm14196731)
Supplement: Supplementary file 1 [file jcm-14-06731-s001.zip › Table S1.pdf]

Table S1. Full Search Strategies

Database: PubMed (MEDLINE)

("non-intubated VATS"[tiab] OR "nonintubated VATS"[tiab] OR "awake VATS"[tiab] OR "tubeless thoracic surgery"[tiab] OR "non-intubated video-assisted thoracoscopic surgery"[tiab] OR "NIVATS"[tiab]) AND ("video-assisted thoracoscopic surgery"[MeSH Terms] OR "VATS"[tiab] OR "thoracoscopic surgery"[tiab]) AND ("lung neoplasms"[MeSH Terms] OR "lung cancer"[tiab] OR "non-small cell lung cancer"[tiab] OR "NSCLC"[tiab])
Filters applied: Humans, English, publication date 2010/01/01 – 2025/06/30

Database: Scopus (Elsevier)

TITLE-ABS-KEY ("non-intubated VATS" OR "awake VATS" OR "tubeless thoracic surgery" OR "NIVATS") AND TITLE-ABS-KEY ("video-assisted thoracoscopic surgery" OR "VATS" OR "thoracoscopic surgery") AND TITLE-ABS-KEY ("lung cancer" OR "NSCLC" OR "non-small cell lung cancer") AND (EXCLUDE (DOCTYPE, "le")) AND (PUBYEAR > 2009 AND PUBYEAR < 2026)

Database: Cochrane CENTRAL

("non-intubated" OR "nonintubated" OR "awake" OR "tubeless") in Title Abstract Keyword AND ("video-assisted thoracoscopic surgery" OR "VATS" OR "thoracoscopic surgery") in Title Abstract Keyword AND ("lung cancer" OR "NSCLC" OR "non-small cell lung cancer") in Title Abstract Keyword
Date range: 2010 – June 2025

Other Sources

• ClinicalTrials.gov and WHO ICTRP were searched with the terms “non-intubated VATS” and “awake VATS.”
• Conference abstracts (AATS, STS, EACTS) were screened manually.
• Reference lists and forward citation searches in Google Scholar (“cited by”) were reviewed for additional eligible studies.
